# Relationship between baseline and changed serum uric acid and the incidence of type 2 diabetes mellitus: a national cohort study

**DOI:** 10.3389/fpubh.2023.1170792

**Published:** 2023-07-06

**Authors:** Congzhi Wang, Jiazhi Wang, Rui Wan, Ting Yuan, Liu Yang, Dongmei Zhang, Xiaoping Li, Min Wang, Haiyang Liu, Yunxiao Lei, Huanhuan Wei, Jing Li, Mingming Liu, Ying Hua, Lu Sun, Lin Zhang

**Affiliations:** ^1^Department of Internal Medicine Nursing, School of Nursing, Wannan Medical College, Higher Education Park, Wuhu, China; ^2^Sports Institute, Chi Zhou College, Education Park, Chi Zhou, China; ^3^Business School, Yunnan University of Finance and Economics, Kunming, China; ^4^Obstetrics and Gynecology Nursing, School of Nursing, Wannan Medical College, Higher Education Park, Wuhu, China; ^5^Department of Pediatric Nursing, School of Nursing, Wannan Medical College, Higher Education Park, Wuhu, China; ^6^Department of Emergency and Critical Care Nursing, School of Nursing, Wannan Medical College, Higher Education Park, Wuhu, China; ^7^Department of Pharmacy, Hainan General Hospital (Hainan Affiliated Hospital of Hainan Medical University), Haikou, China; ^8^Student Health Center, Wannan Medical College, Higher Education Park, Wuhu, China; ^9^Department of Surgical Nursing, School of Nursing, Wannan Medical College, Higher Education Park, Wuhu, China; ^10^Rehabilitation Nursing, School of Nursing, Wannan Medical College, Higher Education Park, Wuhu, China

**Keywords:** type 2 diabetes mellitus, serum uric acid, cohort study, baseline, changes, incidence

## Abstract

**Objective:**

To explore the correlation between baseline serum uric acid (SUA) and SUA changes with the incidence of type 2 diabetes mellitus (T2DM) among middle-aged and older individuals.

**Method:**

Binary logistic regression was used to calculate the odds ratio (ORs) and 95% confidence intervals (CIs) of the effects of baseline and changes in SUA on the incidence of T2DM. Stratified analysis was conducted based on sex, and the SUA levels were classified into four quartiles to assess the effect of baseline and relative changes in SUA on the incidence of T2DM. Furthermore, interaction analysis was performed between body mass index (BMI) and SUA, age and SUA, and sex and SUA.

**Results:**

In the cohort study, the highest quartiles of SUA were significantly correlated with an increased incidence of T2DM among females in model 1 [OR = 2.231 (1.631, 3.050)], model 2 [OR = 2.090 (1.523, 2.867)], model 3 [OR = 2.075 (1.511, 2.849)], and model 4 [OR = 1.707 (1.234, 2.362)]. The highest quartiles of SUA had a statistically significant effect on the incidence of T2DM among all participants in model 1 [OR = 1.601 (1.277, 2.008)], model 2 [OR = 1.519 (1.204, 1.915)], model 3 [OR = 1.597 (1.257, 2.027)], and model 4 [OR = 1.380 (1.083, 1.760)]. Regarding the relative change of SUA, the highest quantiles of SUA were significantly correlated with an increased incidence of T2DM among females in model 1 [OR = 1.409 (1.050, 1.890)], model 2 [OR = 1.433 (1.067, 1.926)], and model 3 [OR = 1.420 (1.056, 1.910)], and there was a statistically significant correlation with incident T2DM among all participants in model 4 [OR = 1.346 (1.079, 1.680)] after adjusting for all covariates. However, there was no significant correlation between baseline, relative, and absolute changes in SUA and the incidence of T2DM among males. The interaction analysis demonstrated that sex, BMI, and the relative changes in SUA had a combined effect on the incidence of T2DM, while age and the changes in SUA had a joint effect on the incidence of T2DM only in females.

**Conclusion:**

There was a positive association between SUA and the incidence of T2DM for all participants. However, significant sex differences in incidence were observed only in women, not men.

## Introduction

Type 2 diabetes mellitus (T2DM) is the most prevalent and chronic disease that has gained substantial attention from the public and healthcare industry over recent decades (1, 2). According to data released by the International Diabetes Federation in 2021, the total number of T2DM cases in China had reached 140 million (3), while 11.3% of the American population has been diagnosed with T2DM (4). In Europe, the prevalence of T2DM was 6.2% among adults in 2019 (5). T2DM contributes to various complications and high mortality rates because of its prevalence, making it the greatest public health challenge globally (6). Thus, multiple potential risk factors have been explored and found to be strongly associated with a high risk of T2DM, including genetic factors, dietary outcomes, obesity, and inactivity, among others (7). Nevertheless, identifying individual risk factors and scientifically demonstrating their risk ratio relationship can beneficially affect the future treatment and prevention of T2DM (8).

Serum uric acid (SUA), a metabolite of purines, is a major enzyme for allantoin conversion, and humans are prone to hyperuricemia when they lack urea oxidase (9). Additionally, a diet rich in high-purine foods tends to lead to high uric acid, contributing to an increasing trend of hyperuricemia (10). In China, the estimated hyperuricemia prevalence ranged from 11.1 to 14.0% in 2015 and 2019, respectively (11). As a catalyst for hyperuricemia, a high concentration of SUA accelerates the progression of insulin resistance and promotes oxidative stress in the extracellular environment. This indicates that an increasing level of SUA can stimulate metabolic imbalance and contribute to diabetes (12). Even though the association between SUA and diabetes as a potential risk factor has been assessed, the conclusion remains debatable owing to the diversity of SUA’s metabolic mechanism (13). The hypothesis has been demonstrated in a meta-analysis of eight well-designed prospective studies, which revealed a positive correlation between SUA and diabetes (14). Non-linear associations, including L-shape, U-shape, inverted U-shape, and bell-shape, were reported in these studies (15–18), making it difficult to establish a definite relationship. Therefore, it remains unclear whether high levels of SUA can influence the incidence of T2DM (19).

Additionally, several previous studies have shown that the association between SUA and T2DM varies based on sex, group, and ethnicity, as demonstrated through stratified analysis (20–22). In China, previous studies have revealed a positive association between SUA and T2DM in evaluating women but not men (23–26). Conversely, a study from Japan indicated that an increasing level of SUA was an independent risk factor in females, while a protective role was observed for males (27). By contrast, Cheng et al. suggested that SUA had slightly protective effects in females but not in males (17). Thus, further research is necessary to clarify the differences in the effects of SUA on sex in Asians, given the varying results reported (8).

Up until now, most studies regarding the relationship between SUA and T2DM have been cross-sectional, making it difficult to identify the potential influence of SUA on T2DM through these observational studies at a single point in time. Although some studies have proven that the accumulation of SUA is strongly associated with T2DM through these cohort studies, a definite association has not been established (2, 25, 28, 29). Additionally, there is a relative paucity of studies examining the impact of changes in SUA on the incidence of T2DM over a relatively long time (2, 25, 28). Thus, recognizing these limitations, we aimed to explore the effects of SUA baseline and the changes in SUA on the incidence of T2DM among middle-aged and older adults from 2011 to 2015 through longitudinal studies based on data extracted from the China Health and Retirement Longitudinal Study (CHARLS). Additionally, this study examined sex differences and performed stratified analyses to investigate the differences in demographic characteristics in this nationwide cohort study.

## Materials and methods

### Participants

**Figure 1:**
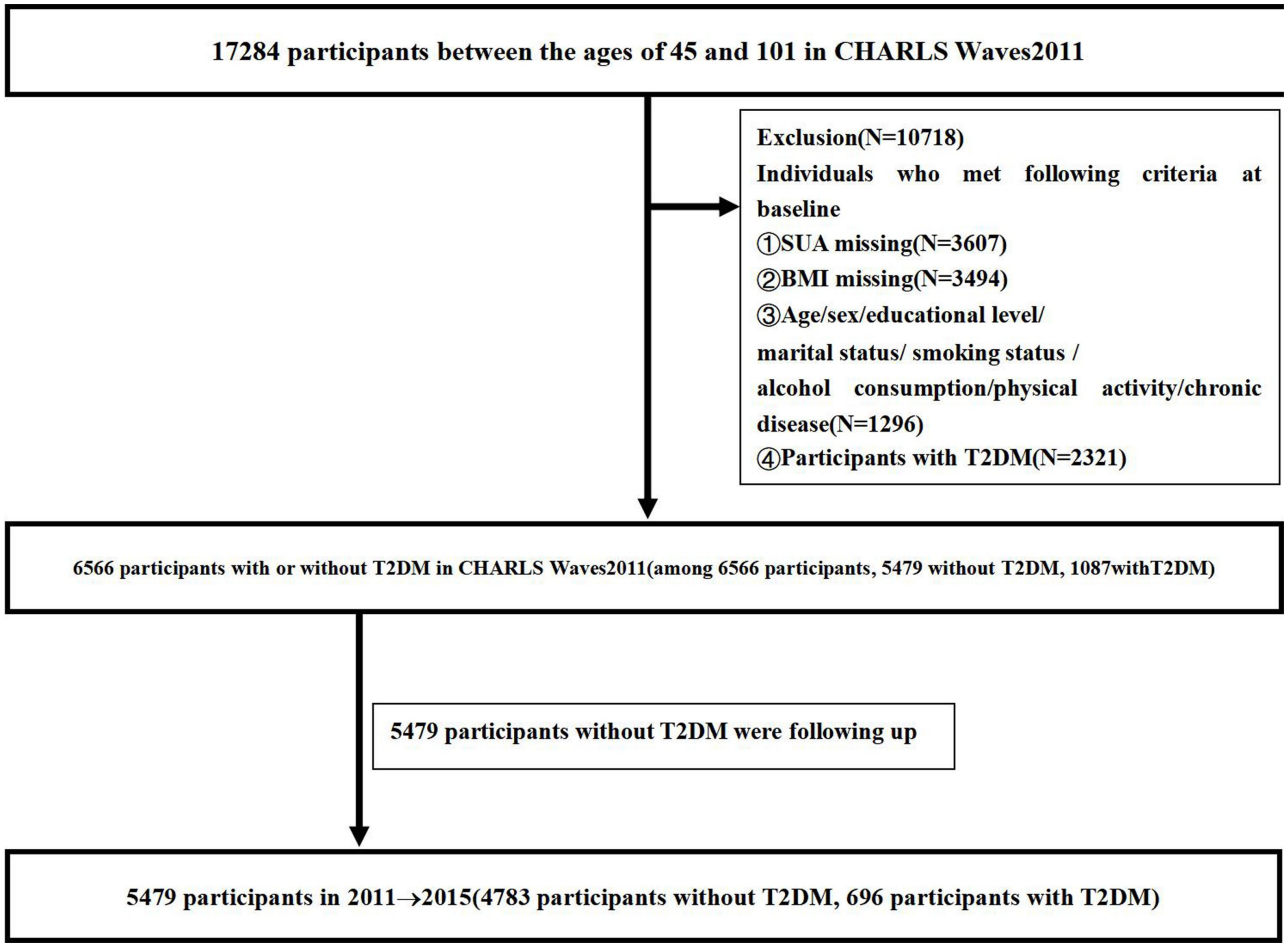
The flowchart of the participants enrolled in the study.

We retrieved data from the CHARLS survey conducted between 2011 and 2015, which is a national longitudinal study administered by the China Center for Economic Research at Peking University. All participants were over 45 years of age, and a structured questionnaire based on a Computer Assisted Personal Interview was used to collect data every 2 years. Data were collected from Waves2011 and Waves2015. Participants without follow-up data and those individuals without data on age, sex, education level, marital status, current residence, smoking status, alcohol consumption, social interaction, physical activity, chronic disease, and BMI were excluded from this study. Additionally, we excluded participants who met any of the following criteria at baseline: (i) had T2DM and (ii) lacked any of the indices of age, sex, education level, marital status, smoking status, alcohol consumption, physical activity, chronic disease, SUA, and BMI. Out of 17,284 participants, we excluded 2,321 with T2DM, 3,607 missing SUA data, 3,494 missing BMI data, and 1,296 missing data on age, sex, education level, marital status, smoking status, alcohol consumption, physical activity, and chronic disease at baseline. In total, 6,566 participants without T2DM from CHARLS Waves2011 were enrolled in the study, among whom 1,087 (16.55%) were diagnosed with T2DM. Thus, we analyzed data from a total of 5,479 participants, including 696 (12.70%) diagnosed with T2DM. Figure 1 shows the flowchart of the study participants, follow-up, and loss to follow-up.

### Diagnostic criteria of T2DM

According to the latest definition of T2DM by the International Diabetes Association, we classified the symptoms based on the diagnostic criteria for T2DM used in previous studies, which are as follows: fasting blood glucose of ≥7.0 mmol/L or HbA1c of ≥6.5% (30–33).

### Measurement of FPG, HbA1c, SUA, and BMI

After obtaining participants’ consent, 10 mL of venous blood was collected from each participant and immediately stored at −20°C (34). Fasting plasma glucose (FPG) and glycated hemoglobin (HbA1c) were analyzed using enzymatic colorimetric tests at the Clinical Laboratory at Capital Medical University in 2011 and 2015 (35). SUA was measured using the ultraviolet plus method, and the normal values for SUA are ≤7.00 mg/dL and ≤ 6.00 mg/dL for adult males and adult females, respectively (36, 37). The relative change in SUA (mg/dL) was calculated as SUA_2015_–SUA_2011_ and the absolute change in SUA was calculated as (SUA_2015_–SUA_2011_)/SUA_2015_. Body mass index (BMI) was used to measure participants’ weight; BMI = weight (kg)/[height (m)]^2^ (33). Height was measured using a metal column height meter (accurate to 0.1 cm), and weight was measured using an electronic weight scale (RGZ-120; accurate to 0.1 kg). Outer clothing and shoes were removed before measuring height and weight.

### Quartile method

The quartile method was applied for the following parameters. First, the baseline SUA levels in 2011 for participants diagnosed with T2DM in 2011 (*N* = 6,566) and 2015 (*N* = 5,479), and the relative and absolute changes in SUA from 2011 to 2015 (*N* = 5,479). Second, after stratified analysis by sex, the baseline SUA levels for males (*N* = 2,962) and females (*N* = 3,604) diagnosed with T2DM in 2011 and the baseline SUA levels for males (*N* = 2,478) and females (*N* = 3,001) diagnosed with T2DM in 2015. Third, after stratified analysis by age, the SUA levels for individuals aged 45–54 years (*N* = 1,921), 55–64 years (*N* = 2,180), 65–74 years (*N* = 1,089), and ≥ 75 years (*N* = 289). Fourth, after stratified analysis by BMI in 2011, the BMI of <18.5 kg/m^2^ (*N* = 342), 18.5–24 kg/m^2^ (*N* = 2,982), 24–28 kg/m^2^ (*N* = 1,563), and ≥ 28 kg/m^2^ (*N* = 592). The cutoff points are presented in Tables 1–10. According to the baseline SUA levels in 2011, and the relative and absolute changes in SUA, the data were divided into four equal parts: quartile 1 (Q1), quartile 2 (Q2), quartile 3 (Q3), and quartile 4 (Q4), representing 0 to 25%, 26 to 50%, 51 to 75%, and 76 to 100% of the SUA index from lowest to highest.

**Table 1:** Baseline characteristics (CHARLS Waves 2011) classified according to the subsequent onset of T2DM in 2011 (*N*, %).

Variables	All participants	Without T2DM	With T2DM	*t/χ2*	*p*-value
*Participants*	6,566	5,479	1,087		
*Age (years)*	58.85 ± 9.02	58.55 ± 9.09	60.34 ± 8.56	7.986	0.005
*Age groups (years)*					
45–54	2,196 (33.45)	1921 (35.06)	275 (25.30)	38.899	0.000
55–64	2,674 (40.72)	2,180 (39.79)	494 (45.45)		
65–74	1,341 (20.42)	1,089 (19.88)	252 (23.18)		
≥75	355 (5.41)	289 (5.27)	66 (6.07)		
**Sex**					
Male	2,962 (45.11)	2,478 (45.23)	484 (44.53)	0.180	0.671
Female	3,604 (54.89)	3,001 (54.77)	603 (55.47)		
*Education level*				3.762	0.288
Illiteracy	1889 (28.77)	1,562 (28.51)	327 (30.08)		
Below primary school	4,109 (62.58)	3,440 (62.78)	669 (61.55)		
Senior high school	397 (6.05)	340 (6.21)	57 (5.24)		
Above technical school	171 (2.60)	137 (2.50)	34 (3.13)		
*Marital status*				0.353	0.563
Single	739 (11.25)	611 (11.15)	128 (11.78)		
Married	5,827 (88.75)	4,868 (88.85)	959 (88.22)		
*Current residence*				4.413	0.039
Countryside	6,157 (93.77)	5,153 (94.05)	1,004 (92.36)		
City	409 (6.23)	326 (5.95)	83 (7.64)		
*Smoking status*					
Non-smoking	4,077 (62.10)	3,398 (62.02)	679 (62.47)	8.457	0.015
Former smoking	542 (8.25)	431 (7.87)	111 (10.21)		
Current smoking	1947 (29.65)	1,650 (30.11)	297 (27.32)		
*Alcohol consumption*				1.728	0.421
No	4,449 (67.76)	3,694 (67.42)	755 (69.46)		
Less	514 (7.83)	434 (7.92)	80 (7.36)		
More	1,603 (24.41)	1,351 (24.66)	252 (23.18)		
*Social interaction*				2.992	0.084
No	3,244 (49.41)	2,733 (49.88)	511 (47.01)		
Yes	3,322 (50.59)	2,746 (50.12)	576 (52.99)		
*Physical activity*					
No	4,012 (61.10)	3,314 (60.49)	698 (64.21)	5.514	0.063
Not regular	1,302 (19.83)	1,108 (20.22)	194 (17.85)		
Regular	1,252 (19.07)	1,057 (19.29)	195 (17.94)		
*Chronic disease groups (counts)*					
0	2028 (30.88)	1817 (33.16)	211 (19.41)	238.792	0.000
1–2	3,284 (50.02)	2,791 (50.94)	493 (45.35)		
3–14	1,254 (19.10)	871 (15.90)	383 (35.24)		
*BMI (kg/m^2^)*	23.66 ± 3.90	23.45 ± 3.80	24.76 ± 4.23	9.202	0.002
*BMI categories*					
<18.5	378 (5.76)	342 (6.24)	36 (3.31)	109.531	0.000
18.5–24	3,440 (52.39)	2,982 (54.43)	458 (42.13)		
24–28	1943 (29.59)	1,563 (28.53)	380 (34.96)		
≥28	805 (12.26)	592 (10.80)	213 (19.60)		
*SUA*					
Q1	1,639 (24.96)	1,381 (25.20)	258 (23.73)	9.657	0.022
Q2	1,646 (25.07)	1,392 (25.41)	254 (23.37)		
Q3	1,644 (25.04)	1,380 (25.19)	264 (24.29)		
Q4	1,637 (24.93)	1,326 (24.20)	311 (28.61)		

CHARLS, China Health and Retirement Longitudinal Study; T2DM, type 2 diabetes mellitus; BMI, body mass index; SUA, serum uric acid. The baseline of SUA in 2011 for those people diagnosed with T2DM in 2011: <3.533 mg/dL, 3.533 to < 4.235 mg/dL, 4.235 to < 5.085 mg/dL, ≥5.085 mg/dL.

**Table 2:** Baseline characteristics (CHARLS Waves 2011) classified according to quartiles of baseline SUA in 2011 (*N*, %).

Variables	All participants	Q_1_	Q2	Q3	Q4	*t/χ2*	*p*-value
Participants	6,566	1,639	1,646	1,644	1,637		
*Age (years)*	58.85 ± 9.02	56.91 ± 8.70	58.44 ± 8.78	59.40 ± 9.05	60.66 ± 9.15	51.584	0.000
*Age groups (years)*						134.41	0.000
45–54	2,196 (33.45)	681 (41.55)	572 (34.75)	502 (30.54)	441 (26.94)		
55–64	2,674 (40.72)	654 (39.90)	680 (41.31)	677 (41.18)	663 (40.50)		
65–74	1,341 (20.42)	246 (15.01)	324 (19.69)	368 (22.38)	403 (24.62)		
≥75	355 (5.41)	58 (3.54)	70 (4.25)	97 (5.90)	130 (7.94)		
*Sex*						951.247	0.000
Male	2,962 (45.11)	329 (20.07)	591 (35.91)	887 (53.95)	1,155 (70.56)		
Female	3,604 (54.89)	1,310 (79.93)	1,055 (64.09)	757 (46.05)	482 (29.44)		
*Education level*						126.889	0.000
Illiteracy	1889 (28.77)	575 (35.08)	519 (31.53)	463 (28.16)	332 (20.28)		
Below primary school	4,109 (62.58)	955 (58.27)	1,003 (60.94)	1,033 (62.83)	1,118 (68.30)		
Senior high school	397 (6.05)	91 (5.55)	91 (5.53)	103 (6.27)	112 (6.84)		
Above technical school	171 (2.60)	18 (1.10)	33 (2.00)	45 (2.74)	75 (4.58)		
*Marital status*						1.779	0.000
Single	739 (11.25)	182 (11.10)	183 (11.12)	199 (12.10)	175 (10.69)		
Married	5,827 (88.75)	1,457 (88.90)	1,463 (88.88)	1,445 (87.90)	1,462 (89.31)		
*Current residence*						32.455	0.000
Countryside	6,157 (93.77)	1,571 (95.85)	1,553 (94.35)	1,541 (93.73)	1,492 (91.14)		
City	409 (6.23)	68 (4.15)	93 (5.65)	103 (6.27)	145 (8.86)		
*Smoking status*						457.536	0.000
Non-smoking	4,077 (62.10)	1,283 (78.28)	1,130 (68.65)	933 (56.75)	731 (44.65)		
Former smoking	542 (8.25)	61 (3.72)	100 (6.08)	152 (9.25)	229 (13.99)		
Current smoking	1947 (29.65)	295 (18.00)	416 (25.27)	559 (34.00)	677 (41.36)		
*Alcohol consumption*						332.912	0.000
No	4,449 (67.76)	1,319 (80.48)	1,195 (72.60)	1,045 (63.56)	890 (54.37)		
Less	514 (7.83)	101 (6.16)	133 (8.08)	156 (9.49)	124 (7.57)		
More	1,603 (24.41)	219 (13.36)	318 (19.32)	443 (26.95)	623 (38.06)		
*Social interaction*						13.332	0.004
No	3,244 (49.41)	854 (52.10)	826 (50.18)	813 (49.45)	751 (45.88)		
Yes	3,322 (50.59)	785 (47.90)	820 (49.82)	831 (50.55)	886 (54.12)		
*Physical activity*						7.457	0.281
No	4,012 (61.10)	966 (58.94)	1,019 (61.91)	1,021 (62.11)	1,006 (61.45)		
Not regular	1,302 (19.83)	359 (21.90)	317 (19.26)	319 (19.40)	307 (18.75)		
Regular	1,252 (19.07)	314 (19.16)	310 (18.83)	304 (18.49)	324 (19.80)		
*Chronic disease groups(counts)*						14.478	0.025
0	2028 (30.89)	541 (33.01)	527 (32.02)	488 (29.68)	472 (28.83)		
1–2	3,284 (50.01)	820 (50.03)	807 (49.03)	839 (51.04)	818 (49.97)		
3–14	1,254 (19.10)	278 (16.96)	312 (18.95)	317 (19.28)	347 (21.20)		
*BMI (kg/m^2^)*	23.66 ± 3.90	23.22 ± 3.63	23.52 ± 3.92	23.81 ± 3.86	24.11 ± 4.12	16.009	0.000
*BMI categories*						51.354	0.000
<18.5	378 (5.76)	115 (7.02)	109 (6.62)	74 (4.50)	80 (4.89)		
18.5–24	3,440 (52.39)	923 (56.32)	867 (52.68)	852 (51.82)	798 (48.75)		
24–28	1943 (29.59)	439 (26.78)	491 (29.83)	503 (30.60)	510 (31.15)		
≥28	805 (12.26)	162 (9.88)	179 (10.87)	215 (13.08)	249 (15.21)		
*T2DM*						9.657	0.022
No	5,479 (83.45)	1,381 (84.26)	1,392 (84.57)	1,380 (83.94)	1,326 (81.00)		
Yes	1,087 (16.55)	258 (15.74)	254 (15.43)	264 (16.06)	311 (19.00)		

CHARLS, China Health and Retirement Longitudinal Study; T2DM, type 2 diabetes mellitus; BMI, body mass index; SUA, serum uric acid. The baseline of SUA in 2011 for those people diagnosed with T2DM in 2011: < 3.533 mg/dL, 3.533 to < 4.235 mg/dL, 4.235 to < 5.085 mg/dL, ≥ 5.085 mg/dL.

**Table 3:** Baseline characteristics (CHARLS Waves 2011) classified according to the subsequent onset of T2DM in 2015 (*N*, %).

Variables	All participants	Without T2DM	With T2DM	*t/χ2*	*p*-value
Participants	5,479	4,783	696		
*Age (years)*	58.56 ± 9.09	58.29 ± 9.03	60.36 ± 9.27	0.535	0.464
*Age groups (years)*				23.826	0.000
45–54	1921 (35.06)	1718 (35.92)	203 (29.17)		
55–64	2,180 (39.79)	1906 (39.85)	274 (39.37)		
65–74	1,089 (19.88)	925 (19.34)	164 (23.56)		
≥75	289 (5.27)	234 (4.89)	55 (7.90)		
**Sex**				0.100	0.935
Male	2,478 (45.23)	2,162 (45.20)	316 (45.40)		
Female	3,001 (54.77)	2,621 (54.80)	380 (54.60)		
*Education level*				5.743	0.125
Illiteracy	1,562 (28.51)	1,357 (28.37)	205 (29.45)		
Below primary school	3,440 (62.78)	2,996 (62.64)	444 (63.79)		
Senior high school	340 (6.21)	311 (6.50)	29 (4.17)		
Above technical school	137 (2.50)	119 (2.49)	18 (2.59)		
*Marital status*				5.614	0.020
Single	611 (11.15)	515 (10.77)	96 (13.79)		
Married	4,868 (88.85)	4,268 (89.23)	600 (86.21)		
*Current residence*				0.100	0.932
Countryside	5,153 (94.05)	4,499 (94.06)	654 (93.97)		
City	326 (5.95)	284 (5.94)	42 (6.03)		
*Smoking status*				0.585	0.746
Non-smoking	3,398 (62.02)	2,975 (62.20)	423 (60.78)		
Former smoking	431 (7.87)	376 (7.86)	55 (7.90)		
Current smoking	1,650 (30.11)	1,432 (29.94)	218 (31.32)		
*Alcohol consumption*				5.379	0.068
No	3,694 (67.42)	3,199 (66.88)	495 (71.12)		
Less	434 (7.92)	389 (8.13)	45 (6.47)		
More	1,351 (24.66)	1,195 (24.99)	156 (22.41)		
*Social interaction*				0.022	0.882
No	2,733 (49.88)	2,384 (49.84)	349 (50.14)		
Yes	2,746 (50.12)	2,399 (50.16)	347 (49.86)		
*Physical activity*				0.281	0.869
No	3,314 (60.49)	2,897 (60.57)	417 (59.91)		
Not regular	1,108 (20.22)	962 (20.11)	146 (20.98)		
Regular	1,057 (19.29)	924 (19.32)	133 (19.11)		
*Chronic disease groups (counts)*				12.694	0.002
0	1817 (33.16)	1,624 (33.95)	193 (27.73)		
1–2	2,791 (50.94)	2,420 (50.60)	371 (53.30)		
3–14	871 (15.90)	739 (15.45)	132 (18.97)		
*BMI (kg/m^2^)*	23.45 ± 3.80	23.28 ± 3.71	24.57 ± 4.21	20.883	0.000
*BMI categories*				74.793	0.000
<18.5	342 (6.24)	310 (6.48)	32 (4.60)		
18.5–24	2,982 (54.43)	2,680 (56.03)	302 (43.39)		
24–28	1,563 (28.53)	1,332 (27.85)	231 (33.19)		
≥28	592 (10.80)	461 (9.64)	131 (18.82)		
*SUA*				18.497	0.000
Q1	1,371 (25.02)	1,228 (25.67)	143 (20.55)		
Q2	1,375 (25.10)	1,212 (25.34)	163 (23.42)		
Q3	1,365 (24.91)	1,190 (24.88)	175 (25.14)		
Q4	1,368 (24.97)	1,153 (24.11)	215 (30.89)		

CHARLS, China Health and Retirement Longitudinal Study; T2DM, type 2 diabetes mellitus; BMI, body mass index; SUA, serum uric acid. The cutoff values baseline of SUA in 2011 for those people diagnosed with T2DM in 2015: <3.524 mg/dL, 3.524 to < 4.224 mg/dL, 4.224 to < 5.055 mg/dL, ≥5.055 mg/dL.

**Table 4:** Baseline characteristics (CHARLS Waves 2011) classified according to quartiles of the subsequent onset of T2DM in 2015 (*N*, %).

Variables	All participants	Q_1_	Q2	Q3	Q4	*t/χ2*	*p*-value
*Participants*	5,479	1,371	1,375	1,365	1,368		
*Age (years)*	58.56 ± 9.09	56.60 ± 8.76	58.18 ± 8.86	59.06 ± 9.15	60.39 ± 9.15	42.805	0.000
*Age groups (years)*						113.785	0.000
45–54	1921 (35.06)	598 (43.62)	505 (36.73)	440 (32.23)	378 (27.63)		
55–64	2,180 (39.79)	523 (38.15)	544 (39.56)	554 (40.59)	559 (40.86)		
65–74	1,089 (19.88)	203 (14.80)	270 (19.64)	288 (21.10)	328 (23.98)		
≥75	289 (5.27)	47 (3.43)	56 (4.07)	83 (6.08)	103 (7.53)		
*Sex*						853.616	0.000.
Male	2,478 (45.23)	264 (19.26)	487 (35.42)	753 (55.16)	974 (71.20)		
Female	3,001 (54.77)	1,107 (80.74)	888 (64.58)	612 (44.84)	394 (28.80)		
*Education level*						109.253	0.000
Illiteracy	1,562 (28.51)	488 (35.60)	432 (31.42)	373 (27.32)	269 (19.67)		
Below primary school	3,440 (62.78)	786 (57.33)	840 (61.09)	868 (63.59)	946 (69.15)		
Senior high school	340 (6.21)	79 (5.76)	78 (5.67)	85 (6.23)	98 (7.16)		
Above technical school	137 (2.50)	18 (1.31)	25 (1.82)	39 (2.86)	55 (4.02)		
*Marital status*							
Single	611 (11.15)	149 (10.87)	157 (11.42)	164 (12.01)	141 (10.31)	2.221	0.528
Married	4,868 (88.85)	1,222 (89.13)	1,218 (88.58)	1,201 (87.99)	1,227 (89.69)		
*Current residence*						23.449	0.000
Countryside	5,153 (94.05)	1,316 (95.99)	1,302 (94.69)	1,280 (93.77)	1,255 (91.74)		
City	326 (5.95)	55 (4.01)	73 (5.31)	85 (6.23)	113 (8.26)		
*Smoking status*						426.219	0.000
Non-smoking	3,398 (62.02)	1,084 (79.07)	953 (69.31)	761 (55.75)	600 (43.86)		
Former smoking	431 (7.87)	47 (3.43)	77 (5.60)	121 (8.87)	186 (13.60)		
Current smoking	1,650 (30.11)	240 (17.50)	345 (25.09)	483 (35.38)	582 (42.54)		
*Alcohol consumption*						299.538	0.000
No	3,694 (67.42)	1,103 (80.45)	1,004 (73.02)	847 (62.05)	740 (54.09)		
Less	434 (7.92)	84 (6.13)	104 (7.56)	146 (10.70)	100 (7.31)		
More	1,351 (24.66)	184 (13.42)	267 (19.42)	372 (27.25)	528 (38.60)		
*Social interaction*						11.376	0.010
No	2,733 (49.88)	721 (52.59)	691 (50.25)	688 (50.40)	633 (46.27)		
Yes	2,746 (50.12)	650 (47.41)	684 (49.75)	677 (49.60)	735 (53.73)		
*Physical activity*						9.307	0.157
No	3,314 (60.49)	795 (57.99)	847 (61.60)	851 (62.34)	821 (60.01)		
Not regular	1,108 (20.22)	308 (22.46)	261 (18.98)	269 (19.71)	270 (19.74)		
Regular	1,057 (19.29)	268 (19.55)	267 (19.42)	245 (17.95)	277 (20.25)		
*Chronic disease groups*						7.036	0.318
0	1817 (33.16)	480 (35.01)	468 (34.04)	432 (31.65)	437 (31.94)		
1–2	2,791 (50.94)	694 (50.62)	682 (49.60)	712 (52.16)	703 (51.39)		
3–14	871 (15.90)	197 (14.37)	225 (16.36)	221 (16.19)	228 (16.67)		
*BMI (kg/m^2^)*	23.45 ± 3.80	23.02 ± 3.57	23.27 ± 3.72	23.63 ± 3.83	23.86 ± 4.00	13.318	0.000
*BMI categories*						42.637	0.000
<18.5	342 (6.24)	103 (7.51)	99 (7.20)	67 (4.91)	73 (5.34)		
18.5–24	2,982 (54.43)	799 (58.28)	755 (54.91)	723 (52.97)	705 (51.53)		
24–28	1,563 (28.53)	352 (25.68)	389 (28.29)	417 (30.55)	405 (29.61)		
≥28	592 (10.80)	117 (8.53)	132 (9.60)	158 (11.57)	185 (13.52)		
*T2DM*						18.497	0.000
No	4,783 (87.30)	1,228 (89.57)	1,212 (88.15)	1,190 (87.18)	1,153 (84.28)		
Yes	696 (12.70)	143 (10.43)	163 (11.85)	175 (12.82)	215 (15.72)		

CHARLS, China Health and Retirement Longitudinal Study; T2DM, type 2 diabetes mellitus; BMI, body mass index; SUA, serum uric acid. The cutoff values baseline of SUA in 2011 for those people diagnosed with T2DM in 2015: <3.524 mg/dL, 3.524 to < 4.224 mg/dL, 4.224 to < 5.055 mg/dL, ≥5.055 mg/dL.

**Table 5:** Baseline characteristics classified according to quartiles of relative changes in SUA (changes from 2015 to 2011) (*N*, %).

Variables	All participants	Q1	Q2	Q3	Q4	*t/χ2*	*p*-value
*Participants*	5,479	1,369	1,371	1,369	1,370		
*Age (years)*	58.56 ± 9.09	59.05 ± 9.09	58.62 ± 9.14	58.11 ± 8.91	58.44 ± 9.18	2.565	0.053
*Age groups (years)*						13.801	0.130
45–54	1921 (35.06)	438 (31.99)	470 (34.28)	512 (37.40)	501 (36.57)		
55–64	2,180 (39.79)	574 (41.93)	550 (40.12)	528 (38.57)	528 (38.54)		
65–74	1,089 (19.88)	274 (20.02)	280 (20.42)	269 (19.65)	266 (19.42)		
≥75	289 (5.27)	83 (6.06)	71 (5.18)	60 (4.38)	75 (5.47)		
**Sex**							
Male	2,478 (45.23)	631 (46.09)	554 (40.41)	586 (42.80)	707 (51.61)	39.008	0.000
Female	3,001 (54.77)	738 (53.91)	817 (59.59)	783 (57.20)	663 (48.39)		
*Education level*						12.523	0.185
Illiteracy	1,562 (28.51)	421 (30.75)	412 (30.05)	371 (27.10)	358 (26.13)		
Below primary school	3,440 (62.78)	839 (61.29)	843 (61.49)	869 (63.48)	889 (64.89)		
Senior high school	340 (6.21)	77 (5.62)	81 (5.91)	97 (7.08)	85 (6.21)		
Above technical school	137 (2.50)	32 (2.34)	35 (2.55)	32 (2.34)	38 (2.77)		
*Marital status*						7.907	0.048
Single	611 (11.15)	171 (12.49)	128 (9.34)	149 (10.88)	163 (11.90)		
Married	4,868 (88.85)	1,198 (87.51)	1,243 (90.66)	1,220 (89.12)	1,207 (88.10)		
*Current residence*						0.688	0.876
Countryside	5,153 (94.05)	1,287 (94.01)	1,291 (94.16)	1,292 (94.38)	1,283 (93.65)		
City	326 (5.95)	82 (5.99)	80 (5.84)	77 (5.62)	87 (6.35)		
*Smoking status*						21.179	0.002
Non-smoking	3,398 (62.02)	823 (60.12)	901 (65.72)	873 (63.77)	801 (58.47)		
Former smoking	431 (7.87)	110 (8.03)	93 (6.78)	114 (8.33)	114 (8.32)		
Current smoking	1,650 (30.11)	436 (31.85)	377 (27.50)	382 (27.90)	455 (33.21)		
*Alcohol consumption*						31.734	0.000
No	3,694 (67.42)	938 (68.52)	946 (69.00)	959 (70.05)	851 (62.12)		
Less	434 (7.92)	94 (6.86)	120 (8.75)	107 (7.82)	113 (8.25)		
More	1,351 (24.66)	337 (24.62)	305 (22.25)	303 (22.13)	406 (29.63)		
*Social interaction*						1.819	0.611
No	2,733 (49.88)	700 (51.13)	667 (48.65)	678 (49.53)	688 (50.22)		
Yes	2,746 (50.12)	669 (48.87)	704 (51.35)	691 (50.47)	682 (49.78)		
*Physical activity*						8.434	0.208
No	3,314 (60.49)	828 (60.48)	834 (60.83)	814 (59.46)	838 (61.17)		
Not regular	1,108 (20.22)	262 (19.14)	258 (18.82)	298 (21.77)	290 (21.17)		
Regular	1,057 (19.29)	279 (20.38)	279 (20.35)	257 (18.77)	242 (17.66)		
*Chronic disease groups (counts)*						3.229	0.780
0	1817 (33.16)	452 (33.02)	452 (32.97)	467 (34.11)	446 (32.55)		
1–2	2,791 (50.94)	698 (50.98)	717 (52.30)	679 (49.60)	697 (50.88)		
3–14	871 (15.90)	219 (16.00)	202 (14.73)	223 (16.29)	227 (16.57)		
*BMI (kg/m^2^)*	23.45 ± 3.80	23.16 ± 3.71	23.49 ± 3.92	23.36 ± 3.68	23.77 ± 3.84	6.241	0.000
*BMI categories*						24.186	0.004
<18.5	342 (6.24)	90 (6.57)	92 (6.71)	99 (7.23)	61 (4.45)		
18.5–24	2,982 (54.43)	784 (57.27)	737 (53.76)	740 (54.06)	721 (52.63)		
24–28	1,563 (28.53)	370 (27.03)	396 (28.88)	385 (28.12)	412 (30.07)		
≥28	592 (10.80)	125 (9.13)	146 (10.65)	145 (10.59)	176 (12.85)		
*T2DM*						16.402	0.001
No	4,783 (87.30)	1,205 (88.02)	1,214 (88.55)	1,211 (88.46)	1,153 (84.16)		
Yes	696 (12.70)	164 (11.98)	157 (11.45)	158 (11.54)	217 (15.84)		

T2DM, type 2 diabetes mellitus; BMI, body mass index; SUA, serum uric acid. The cutoff values of relative changes in SUA from 2011 to 2015: <−0.066 mg/dL, −0.066 to < 0.487 mg/dL, 0.487 to < 1.094 mg/dL, ≥1.094 mg/dL.

**Table 6:** Baseline characteristics classified according to quartiles of absolute changes in SUA (changes from 2015 to 2011) (*N*, %).

Variables	All participants	Q1	Q2	Q3	Q4	*t/χ2*	*p*-value
*Participants*	5,479	1,370	1,370	1,369	1,370		
*Age (years)*	58.56 ± 9.09	59.07 ± 9.08	58.96 ± 9.13	58.37 ± 8.91	57.82 ± 9.17	5.584	0.001
*Age groups (years)*						24.413	0.004
45–54	1921 (35.06)	437 (31.90)	449 (32.77)	497 (36.30)	538 (39.27)		
55–64	2,180 (39.79)	573 (41.82)	554 (40.44)	533 (38.93)	520 (37.96)		
65–74	1,089 (19.88)	278 (20.29)	288 (21.02)	278 (20.31)	245 (17.88)		
≥75	289 (5.27)	82 (5.99)	79 (5.77)	61 (4.46)	67 (4.89)		
**Sex**						2.008	0.571
Male	2,478 (45.23)	628 (45.84)	636 (46.42)	611 (44.63)	603 (44.01)		
Female	3,001 (54.77)	742 (54.16)	734 (53.58)	758 (55.37)	767 (55.99)		
*Education level*						9.291	0.411
Illiteracy	1,562 (28.51)	421 (30.73)	395 (28.83)	369 (26.95)	377 (27.52)		
Below primary school	3,440 (62.78)	841 (61.39)	850 (62.04)	875 (63.92)	874 (63.79)		
Senior high school	340 (6.21)	77 (5.62)	83 (6.06)	94 (6.87)	86 (6.28)		
Above technical school	137 (2.50)	31 (2.26)	42 (3.07)	31 (2.26)	33 (2.41)		
*Marital status*						9.958	0.019
Single	611 (11.15)	173 (12.63)	125 (9.12)	148 (10.81)	165 (12.04)		
Married	4,868 (88.85)	1,197 (87.37)	1,245 (90.88)	1,221 (89.19)	1,205 (87.96)		
*Current residence*						0.800	0.850
Countryside	5,153 (94.05)	1,288 (94.01)	1,285 (93.80)	1,285 (93.86)	1,295 (94.53)		
City	326 (5.95)	82 (5.99)	85 (6.20)	84 (6.14)	75 (5.47)		
*Smoking status*						3.922	0.687
Non-smoking	3,398 (62.02)	823 (60.07)	853 (62.26)	857 (62.60)	865 (63.14)		
Former smoking	431 (7.87)	110 (8.03)	112 (8.18)	109 (7.96)	100 (7.30)		
Current smoking	1,650 (30.11)	437 (31.90)	405 (29.56)	403 (29.44)	405 (29.56)		
*Alcohol consumption*						5.284	0.508
No	3,694 (67.42)	938 (68.47)	914 (66.71)	940 (68.66)	902 (65.84)		
Less	434 (7.92)	95 (6.93)	116 (8.47)	105 (7.67)	118 (8.61)		
More	1,351 (24.66)	337 (24.60)	340 (24.82)	324 (23.67)	350 (25.55)		
*Social interaction*						3.316	0.345
No	2,733 (49.88)	701 (51.17)	656 (47.88)	684 (49.96)	692 (50.51)		
Yes	2,746 (50.12)	669 (48.83)	714 (52.12)	685 (50.04)	678 (49.49)		
*Physical activity*						12.13	0.059
No	3,314 (60.49)	828 (60.44)	841 (61.39)	807 (58.95)	838 (61.17)		
Not regular	1,108 (20.22)	263 (19.20)	253 (18.47)	291 (21.25)	301 (21.97)		
Regular	1,057 (19.29)	279 (20.36)	276 (20.14)	271 (19.80)	231 (16.86)		
*Chronic disease groups (counts)*						3.406	0.756
0	1817 (33.16)	452 (32.99)	438 (31.97)	458 (33.46)	469 (34.23)		
1–2	2,791 (50.94)	701 (51.17)	720 (52.56)	697 (50.91)	673 (49.13)		
3–14	871 (15.90)	217 (15.84)	212 (15.47)	214 (15.63)	228 (16.64)		
*BMI (kg/m^2^)*	23.45 ± 3.80	23.13 ± 3.69	23.62 ± 3.91	23.39 ± 3.77	23.64 ± 3.80	5.487	0.001
*BMI categories*						17.105	0.047
<18.5	342 (6.24)	92 (6.72)	87 (6.35)	93 (6.79)	70 (5.11)		
18.5–24	2,982 (54.43)	786 (57.37)	718 (52.41)	743 (54.27)	735 (53.65)		
24–28	1,563 (28.53)	370 (27.01)	412 (30.07)	383 (27.98)	398 (29.05)		
≥28	592 (10.80)	122 (8.90)	153 (11.17)	150 (10.96)	167 (12.19)		
*T2DM*						7.433	0.059
No	4,783 (87.30)	1,207 (88.10)	1,203 (87.81)	1,206 (88.09)	1,167 (85.18)		
Yes	696 (12.70)	163 (11.90)	167 (12.19)	163 (11.91)	203 (14.82)		

T2DM, type 2 diabetes mellitus; BMI, body mass index; SUA, serum uric acid. The cutoff values of absolute changes in SUA from 2011 to 2015: <−0.015, 0.015 to 0.118, 0.118 to 0.274, ≥0.274.

**Table 7:** Odds ratios (ORs) and 95% confidence intervals (CIs) for 2011 baseline SUA associated with the prevalence of T2DM in 2011.

N = 6,566	Model 1 OR (95% CI)	Wald, df	*p*	Model 2 OR (95% CI)	Wald, df	*p*	Model 3 OR (95% CI)	Wald, df	*p*	Model 4 OR (95% CI)	Wald, df	*p*
*Male (N = 2,962)*												
Q1 (741)	Ref (1.000)			Ref (1.000)			Ref (1.000)			Ref (1.000)		
Q2 (744)	0.947 (0.719,1.248)	0.1491	0.699	0.943 (0.716,1.243)	0.1721	0.679	0.904 (0.681,1.198)	0.4941	0.482	0.860 (0.647,1.144)	1.0681	0.301
Q3 (737)	0.863 (0.651,1.143)	1.0591	0.303	0.857 (0.647,1.137)	1.1451	0.285	0.827 (0.620,1.103)	1.6671	0.197	0.759 (0.567,1.017)	3.4161	0.065
Q4 (740)	1.121 (0.857,1.467)	0.6951	0.404	1.104 (0.843,1.447)	0.5161	0.473	1.019 (0.772,1.346)	0.0191	0.892	0.883 (0.665,1.174)	0.7321	0.392
*p*-trend	1.028 (0.942,1.121)	0.3771	0.539	1.023 (0.937,1.116)	0.2501	0.617	0.999 (0.913,1.093)	0.0001	0.987	0.949 (0.866,1.041)	1.2271	0.268
*Female (N = 3,604)*												
Q1 (903)	Ref (1.000)			Ref (1.000)			Ref (1.000)			Ref (1.000)		
Q2 (899)	0.907 (0.694,1.186)	0.5081	0.476	0.872 (0.666,1.142)	0.9911	0.319	0.858 (0.654,1.126)	1.2161	0.270	0.827 (0.629,1.087)	1.8541	0.173
Q3 (901)	0.967 (0.742,1.259)	0.0631	0.802	0.916 (0.702,1.196)	0.4151	0.520	0.895 (0.684,1.170)	0.661	0.417	0.840 (0.641,1.101)	1.5901	0.207
Q4 (901)	1.383 (1.077,1.775)	6.4711	0.011	1.237 (0.960,1.596)	2.6951	0.101	1.174 (0.908,1.518)	1.5041	0.220	1.038 (0.799,1.350)	0.0801	0.778
*p*-trend	1.136 (1.050,1.229)	10.1101	0.001	1.099 (1.015,1.190)	5.3881	0.020	1.075 (0.992,1.166)	3.0981	0.078	1.033 (0.951,1.122)	0.5981	0.439
*Total (N = 6,566)*												
Q1 (1639)	Ref (1.000)			Ref (1.000)			Ref (1.000)			Ref (1.000)		
Q2 (1646)	0.977 (0.809,1.179)	0.061	0.807	0.950 (0.786,1.148)	0.2821	0.595	0.939 (0.774,1.138)	0.4131	0.520	0.903 (0.744,1.097)	1.0551	0.304
Q3 (1644)	1.024 (0.849,1.235)	0.0621	0.804	0.977 (0.809,1.180)	0.0601	0.807	0.962 (0.792,1.168)	0.1561	0.693	0.883 (0.725,1.074)	1.5581	0.212
Q4 (1637)	1.255 (1.047,1.505)	6.0381	0.014	1.169 (0.970,1.408)	2.6931	0.101	1.149 (0.945,1.396)	1.9441	0.163	1.012 (0.830,1.234)	0.0141	0.906
*p*-trend	1.078 (1.017,1.143)	6.4001	0.011	1.074 (1.007,1.144)	4.7671	0.029	1.049 (0.983,1.119)	2.0511	0.152	1.000 (0.936,1.068)	0.0001	0.999

ORs, odds ratios; CIs, confidence intervals; SUA, serum uric acid; T2DM, type 2 diabetes mellitus; BMI, body mass index.

Model 1: unadjusted.

Model 2: adjusted for age, sex (total subgroup), education level, marital status, and current residence.

Model 3: adjusted for age, sex (total subgroup), education level, marital status, current residence, smoking status, alcohol consumption, social interaction, physical activity, and chronic disease.

Model 4: adjusted for age, sex (total subgroup), education level, marital status, current residence, smoking status, alcohol consumption, social interaction, physical activity, chronic disease, and BMI.

The cutoff values of baseline SUA in males: <4.051 mg/dL, 4.051 to < 4.759 mg/dL, 4.759 to < 5.648 mg/dL, ≥5.648 mg/dL.

The cutoff values of baseline SUA in females: <3.251 mg/dL, 3.251 to < 3.833 mg/dL, 3.833 to < 4.853 mg/dL, ≥4.853 mg/dL.

The cutoff values of baseline SUA in total participants: <3.533 mg/dL, 3.533 to < 4.235 mg/dL, 4.235 to < 5.085 mg/dL, ≥5.085 mg/dL.

**Table 8:** Odds ratios (ORs) and 95% confidence intervals (CIs) for 2011 baseline SUA associated with the incidence of T2DM in 2015.

*N* = 5,479	Model 1 OR (95% CI)	Wald, df	*p*	Model 2 OR (95% CI)	Wald, df	*p*	Model 3 OR (95% CI)	Wald, df	*p*	Model 4 OR (95% CI)	Wald, df	*p*
*Male (N = 2,478)*												
Q1 (619)	Ref (1.000)			Ref (1.000)			Ref (1.000)			Ref (1.000)		
Q2 (620)	0.978 (0.696,1.374)	0.0171	0.897	0.968 (0.689,1.362)	0.0341	0.854	0.963 (0.684,1.355)	0.0481	0.827	0.904 (0.641,1.276)	0.3291	0.566
Q3 (620)	0.983 (0.699,1.382)	0.0091	0.922	0.967 (0.687,1.361)	0.0371	0.848	0.981 (0.696,1.384)	0.0111	0.915	0.902 (0.637,1.277)	0.3381	0.561
Q4 (619)	1.213 (0.874,1.684)	1.3351	0.248	1.178 (0.847,1.639)	0.9451	0.331	1.197 (0.859,1.669)	1.1271	0.288	1.062 (0.757,1.488)	0.1211	0.728
*P*-trend	1.063 (0.956,1.182)	1.2801	0.258	1.053 (0.946,1.171)	0.8971	0.344	1.060 (0.952,1.180)	1.1431	0.285	1.021 (0.915,1.139)	0.1391	0.709
*Female (N = 3,001)*												
Q1 (749)	Ref (1.000)			Ref (1.000)			Ref (1.000)			Ref (1.000)		
Q2 (752)	1.213 (0.861,1.708)	1.2171	0.270	1.190 (0.844,1.678)	0.9861	0.321	1.187 (0.841,1.675)	0.9511	0.329	1.128 (0.797,1.595)	0.4621	0.497
Q3 (750)	1.577 (1.137,2.189)	7.4421	0.006	1.523 (1.095,2.117)	6.2621	0.012	1.504 (1.081,2.092)	5.8571	0.016	1.370 (0.981,1.913)	3.4091	0.065
Q4 (750)	2.231 (1.631,3.050)	25.2711	0.000	2.090 (1.523,2.867)	20.8781	0.000	2.075 (1.511,2.849)	20.3531	0.000	1.707 (1.234,2.362)	10.4361	0.001
*P*-trend	1.314 (1.191,1.450)	29.4371	0.000	1.286 (1.164,1.420)	24.4021	0.000	1.282 (1.160,1.417)	23.6811	0.000	1.201 (1.085,1.331)	12.3691	0.000
*Total (N = 5,479)*												
Q1 (1371)	Ref (1.000)			Ref (1.000)			Ref (1.000)			Ref (1.000)		
Q2 (1375)	1.155 (0.910,1.466)	1.4051	0.236	1.127 (0.887,1.431)	0.9591	0.328	1.142 (0.898,1.451)	1.171	0.279	1.090 (0.856,1.388)	0.4881	0.485
Q3 (1365)	1.263 (0.998,1.597)	3.7921	0.051	1.211 (0.955,1.535)	2.4941	0.114	1.247 (0.981,1.586)	3.2491	0.071	1.117 (0.876,1.424)	0.7961	0.372
Q4 (1368)	1.601 (1.277,2.008)	16.6341	0.000	1.519 (1.204,1.915)	12.4651	0.000	1.597 (1.257,2.027)	14.7391	0.000	1.380 (1.083,1.760)	6.7651	0.009
*P*-trend	1.165 (1.084,1.251)	17.3841	0.000	1.175 (1.086,1.271)	16.1671	0.000	1.177 (1.088,1.274)	16.4771	0.000	1.112 (1.026,1.205)	6.7161	0.010

ORs, odds ratios; CIs, confidence intervals; SUA, serum uric acid; T2DM, type 2 diabetes mellitus; BMI, body mass index.

Model 1: unadjusted.

Model 2: adjusted for age, sex (total subgroup), education level, marital status, and current residence.

Model 3: adjusted for age, sex (total subgroup), education level, marital status, current residence, smoking status, alcohol consumption, social interaction, physical activity, and chronic disease.

Model 4: adjusted for age, sex (total subgroup), education level, marital status, current residence, smoking status, alcohol consumption, social interaction, physical activity, chronic disease, and BMI.

The cutoff values of baseline SUA in males: <4.054 mg/dL, 4.054 to < 4.758 mg/dL, 4.758 to < 5.625 mg/dL, ≥5.625 mg/dL.

The cutoff values of baseline SUA in females: <3.241 mg/dL, 3.241 to < 3.812 mg/dL, 3.812 to < 4.499 mg/dL, ≥4.499 mg/dL.

The cutoff values of baseline SUA in total participants: <3.524 mg/dL, 3.524 to < 4.224 mg/dL, 4.224 to < 5.055 mg/dL, ≥5.055 mg/dL.

**Table 9:** Odds ratios (ORs) and 95% confidence intervals (Cis) for relative changes of SUA (2011–2015) associated with the incidence of T2DM in 2015.

N = 2,579	Model 1 OR (95% CI)	Wald, df	*p*	Model 2 OR (95% CI)	Wald, df	*p*	Model 3 OR (95% CI)	Wald, df	*p*	Model 4 OR (95% CI)	Wald, df	*p*
*Male (N = 2,478)*												
Q1 (619)	Ref (1.000)			Ref (1.000)			Ref (1.000)			Ref (1.000)		
Q2 (620)	0.923 (0.653,1.306)	0.2041	0.651	0.928 (0.656,1.313)	0.1801	0.671	0.935 (0.660,1.324)	0.1451	0.704	0.918 (0.647,1.303)	0.2291	0.632
Q3 (620)	1.075 (0.767,1.505)	0.1751	0.676	1.088 (0.776,1.524)	0.2391	0.625	1.091 (0.778,1.530)	0.2561	0.613	1.081 (0.770,1.518)	0.2021	0.653
Q4 (619)	1.250 (0.900,1.736)	1.7761	0.183	1.271 (0.914,1.766)	2.0341	0.154	1.312 (0.942,1.827)	2.5841	0.108	1.282 (0.919,1.788)	2.1341	0.144
*P*-trend	1.088 (0.979,1.210)	2.4381	0.118	1.094 (0.984,1.217)	2.7551	0.097	1.104 (0.992,1.229)	3.3091	0.069	1.097 (0.986,1.222)	2.8791	0.090
*Female (N = 3,001)*												
Q1 (749)	Ref (1.000)			Ref (1.000)			Ref (1.000)			Ref (1.000)		
Q2 (752)	0.946 (0.690,1.295)	0.1221	0.727	0.960 (0.699,1.317)	0.0641	0.800	0.964 (0.702,1.325)	0.0501	0.823	0.949 (0.688,1.307)	0.1041	0.747
Q3 (750)	0.911 (0.664,1.251)	0.3311	0.565	0.940 (0.683,1.292)	0.1471	0.702	0.938 (0.681,1.291)	0.1551	0.693	0.936 (0.678,1.293)	0.1611	0.688
Q4 (750)	1.409 (1.050,1.890)	5.2201	0.022	1.433 (1.067,1.926)	5.7011	0.017	1.420 (1.056,1.910)	5.3831	0.020	1.316 (0.974,1.776)	3.2071	0.073
*P*-trend	1.115 (1.012,1.228)	4.8581	0.028	1.122 (1.018,1.236)	5.4101	0.020	1.118 (1.014,1.232)	5.0351	0.025	1.092 (0.991,1.205)	3.1301	0.077
*Total (N = 5,479)*												
Q1 (1371)	Ref (1.000)			Ref (1.000)			Ref (1.000)			Ref (1.000)		
Q2 (1375)	0.950 (0.753,1.199)	0.1851	0.667	0.964 (0.763,1.218)	0.0951	0.758	0.964 (0.763,1.218)	0.0951	0.758	0.948 (0.749,1.200)	0.1971	0.657
Q3 (1365)	0.959 (0.760,1.210)	0.1271	0.722	0.979 (0.775,1.236)	0.0321	0.857	0.979 (0.775,1.236)	0.0321	0.857	0.975 (0.771,1.234)	0.0441	0.835
Q4 (1368)	1.383 (1.112,1.720)	8.4711	0.004	1.405 (1.129,1.749)	9.6821	0.002	1.417 (1.138,1.765)	9.6821	0.002	1.346 (1.079,1.680)	6.9201	0.009
*p*-trend	1.111 (1.035,1.194)	8.3931	0.004	1.118 (1.041,1.201)	9.3371	0.002	1.120 (1.042,1.203)	9.5641	0.002	1.103 (1.026,1.186)	7.1021	0.008

ORs, odds ratios; CIs, confidence intervals; SUA, serum uric acid; T2DM, type 2 diabetes mellitus; BMI, body mass index.

Model 1: unadjusted.

Model 2: adjusted for age, sex (total subgroup), education level, marital status, and current residence.

Model 3: adjusted for age, sex (total subgroup), education level, marital status, current residence, smoking status, alcohol consumption, social interaction, physical activity, and chronic disease.

Model 4: adjusted for age, sex (total subgroup), education level, marital status, current residence, smoking status, alcohol consumption, social interaction, physical activity, chronic disease, and BMI.

The cutoff values of relative changes of SUA in males: <−0.089 mg/dL, −0.089 to < 0.537 mg/dL, 0.537 to < 1.238 mg/dL, ≥1.238 mg/dL.

The cutoff values of relative changes of SUA in females: <−0.058 mg/dL, −0.058 to < 0.455 mg/dL, 0.455 to < 1.005 mg/dL, ≥1.005 mg/dL.

The cutoff values of relative changes of SUA in total participants: <−0.066 mg/dL, −0.066 to < 0.487 mg/dL, 0.487 to < 1.094 mg/dL, ≥1.094 mg/dL.

**Table 10:** Adjusted odds ratios (ORs) and confidence intervals (CIs) for relative and absolute changes of SUA (2011–2015) stratified by age and BMI.

Subgroups	*N*	Quartiles of relative changes in SUA				Quartiles of absolute changes in SUA			
*N* = 5,479		Q1	Q2	Q3	Q4	Q1	Q2	Q3	Q4
*Age group*									
45–54	1921	Ref (1.000)	0.805 (0.522,1.242)	0.993 (0.655,1.504)	1.045 (0.698,1.564)	Ref (1.000)	0.884 (0.58,1.347)	0.894 (0.585,1.366)	1.073 (0.716,1.606)
55–64	2,180	Ref (1.000)	0.854 (0.584,1.248)	0.829 (0.566,1.213)	1.412 (0.996,2.003)	Ref (1.000)	0.828 (0.568,1.207)	0.901 (0.623,1.304)	1.210 (0.850,1.723)
65–74	1,089	Ref (1.000)	1.665 (1.014,2.735)^*^	1.207 (0.718,2.029)	1.760 (1.078,2.873)^*^	Ref (1.000)	1.629 (0.984,2.697)	1.257 (0.745,2.121)	1.949 (1.194,3.180)^*^
≥75	289	Ref (1.000)	0.491 (0.192,1.255)	1.192 (0.509,2.791)	0.687 (0.283,1.668)	Ref (1.000)	0.463 (0.180,1.191)	1.083 (0.464,2.525)	0.832 (0.350,1.974)
*BMI-2011*									
<18.5	342	Ref (1.000)	1.373 (0.489,3.857)	0.761 (0.242,2.39)	0.900 (0.302,2.678)	Ref (1.000)	1.198 (0.418,3.432)	0.681 (0.217,2.136)	1.216 (0.422,3.508)
18.5–24	2,982	Ref (1.000)	0.874 (0.611,1.250)	0.933 (0.657,1.325)	1.451 (1.049,2.003)^*^	Ref (1.000)	0.893 (0.628,1.271)	0.997 (0.707,1.406)	1.301 (0.937,1.805)
24–28	1,563	Ref (1.000)	0.917 (0.610,1.378)	0.960 (0.639,1.442)	1.258 (0.853,1.856)	Ref (1.000)	1.027 (0.687,1.535)	0.968 (0.644,1.457)	1.241 (0.836,1.842)
≥28	592	Ref (1.000)	0.717 (0.402,1.278)	0.955 (0.549,1.661)	1.058 (0.613,1.826)	Ref (1.000)	0.825 (0.469,1.448)	0.895 (0.512,1.565)	1.004 (0.578,1.743)

^*^p < 0.05.

ORs, odds ratios; CIs, confidence; SUA, serum uric acid; BMI, body mass index.

Adjusted for age (not included in the age group analysis), sex (not included in the sex group analysis), education level, marital status, current residence, smoking status, alcohol consumption, social interaction, physical activity, chronic disease, and BMI-2011 (not included in the BMI-2011 group analysis).

The cutoff values of changes in SUA in 45–54 years: relative changes: <−0.006 mg/dL, −0.006 to < 0.540 mg/dL, 0.540 to < 1.129 mg/dL, ≥1.129 mg/dL. Absolute changes: <−0.001, −0.001 to < 0.138, 0.138 to < 0.293, ≥0.293.

The cutoff values of changes in SUA in 55–64 years: relative changes: <−0.108 mg/dL, −0.108 to < 0.451 mg/dL, 0.451 to < 1.067 mg/dL, ≥1.067 mg/dL. absolute changes: <−0.024, −0.024 to < 0.109, 0.109 to < 0.265, ≥0.265.

The cutoff values of changes in SUA in 65–74 years: relative changes: <−0.069 mg/dL, −0.069 to < 0.472 mg/dL, 0.472 to < 1.073 mg/dL, ≥1.073 mg/dL. absolute changes: <−0.018, −0.018 to < 0.107, 0.107 to < 0.255, ≥0.255.

The cutoff values of changes in SUA in ≥ 75 years: relative changes: <−0.167 mg/dL, −0.167 to < 0.393 mg/dL, 0.393 to < 1.109 mg/dL, ≥1.109 mg/dL. absolute changes: <−0.035, −0.035 to < 0.080, 0.080 to < 0.260, ≥0.260.

The cutoff values of changes in SUA in BMI < 18.5 kg/m^2^: relative changes: <−0.088 mg/dL, −0.088 to < 0.432 mg/dL, 0.432 to < 0.874 mg/dL, ≥0.874 mg/dL. absolute changes: <−0.022, −0.022 to < 0.106, 0.106 to < 0.233, ≥0.233.

The cutoff values of changes in SUA in BMI 18.5-24 kg/m^2^: relative changes: <−0.110 mg/dL, −0.110 to < 0.470 mg/dL, 0.470 to < 1.065 mg/dL, ≥1.065 mg/dL. absolute changes: <−0.026, −0.026 to < 0.116, 0.116 to < 0.270, ≥0.270.

The cutoff values of changes in SUA in BMI 24-28 kg/m^2^: relative changes: <−0.040 mg/dL, −0.040 to < 0.509 mg/dL, 0.509 to < 1.313 mg/dL, ≥1.313 mg/dL. absolute changes: <−0.009, −0.009 to < 0.118, 0.118 to < 0.279, ≥0.279.

The cutoff values of changes in SUA in BMI ≥ 28 kg/m^2^: relative changes: <0.013 mg/dL, 0.013 to < 0.582 mg/dL, 0.582 to < 1.308 mg/dL, ≥1.308 mg/dL. absolute changes: <0.002, 0.002 to < 0.136, 0.136 to < 0.307, ≥ 0.307.

### Covariates

The participants were classified into two groups based on whether they had been diagnosed with T2DM or not. Additionally, we included age, sex, education level, marital status, current residence, smoking status, alcohol consumption, social interaction, physical activity, chronic diseases, BMI in 2011, and SUA levels as covariates based on the previous studies (34, 35, 38, 39). The age groups (years) were divided into 45–54 years, 55–64 years, 65–74 years, and ≥ 75 years. Sex was categorized as male and female. Education level was grouped into illiteracy, below primary school, senior high school, and above technical school. Marital status was categorized as single and married. Current residence was divided into countryside and city. Smoking status was categorized as non-smoking, former smoking, and current smoking. Alcohol consumption was classified as none, less, or more. Social interaction was categorized into no or yes. Physical activity was grouped as none, not regular, or regular. Chronic disease groups were categorized into 0, 1–2, and 3–14. BMI categories were classified as <18.5 kg/m^2^, 18.5–24 kg/m^2^, 24–28 kg/m^2^, and ≥ 28 kg/m^2^. The SUA levels at baseline in 2011 and the relative and absolute changes of SUA were categorized into Q1, Q2, Q3, and Q4.

### Statistical analysis

For continuous data with normal distribution, data are presented as mean and standard deviation (SD), while data are presented as frequencies and percentages for categorical data. A Kolmogorov–Smirnov test was used to testify the normality of the data. We compared the differences in basic characteristics using the analysis of variance and chi-square test for continuous and categorical parameters, respectively. Binary logistic regression was used to compute the odds ratio (ORs) and 95% confidence intervals (CIs) of the effects of baseline and changes in SUA on the incidence of T2DM. SPSS 25.0 was used to analysis the data and *p* < 0.05 suggested statistical difference. In model 1, we did not adjust for any covariates. In model 2, we adjusted for age, sex (total subgroup), education level, marital status, and current residence. In model 3, we adjusted for age, sex (total subgroup), education level, marital status, current residence, smoking status, alcohol consumption, social interaction, physical activity, and chronic disease. In model 4, we adjusted for age, sex (total subgroup), education level, marital status, current residence, smoking status, alcohol consumption, social interaction, physical activity, chronic disease, and BMI. In model a, we did not adjust for any covariates. In model b, we adjusted for age, education level, marital status, and current residence. In model c, we adjusted for age, education level, marital status, current residence, smoking status, alcohol consumption, social interaction, and physical activity. In model d, we adjusted for age, education level, marital status, current residence, smoking status, alcohol consumption, social interaction, physical activity, and chronic disease. In model e, we adjusted for education level, marital status, and current residence. In model f, we adjusted for education level, marital status, current residence, smoking status, alcohol consumption, social interaction, physical activity, and chronic disease. In model g, we adjusted for education level, marital status, current residence, smoking status, alcohol consumption, social interaction, physical activity, chronic disease, and BMI. In model h, we adjusted for age, education level, marital status, current residence, smoking status, alcohol consumption, social interaction, physical activity, chronic disease, and BMI. Based on the level of SUA, the quartile method was used to explore the relationship between baseline SUA, changes in SUA, and the incidence of T2DM. Additionally, the stratified method was used to assess the potential difference in demographic factors, including age and BMI-2011. In models 1, 2, 3, and 4, the lowest quartiles of SUA baseline were used as a reference, and the lowest quartile of SUA in each subgroup was used as the reference in subgroup analyses.

## Results

The baseline characteristics of participants according to the subsequent onset of T2DM in 2011 are presented in Table 1. The total number of participants was 6,566, and 5,479 (83.45%) were without T2DM and 1,087 (16.55%) had T2DM. Significant differences were observed in age, current residence, smoking status, chronic disease groups, BMI, and SUA (*p* < 0.05). However, sex, education level, marital status, alcohol consumption, social interaction, and physical activity did not show statistically significant differences between the two groups (with and without T2DM) (*p* > 0.05).

Table 2 shows the baseline characteristics across quartiles of baseline SUA in 2011. The total number of participants was 6,566, and there were 1,639, 1,646, 1,644, and 1,637 in Q1 to Q4, respectively. Significant differences were found in all groups for baseline characteristics except for physical activity (*p* > 0.05).

Table 3 presents the baseline characteristics classified based on the onset of T2DM in 2015. The total number of participants was 5,479, and 4,783 (87.30%) were without T2DM and 696 (12.70%) had T2DM. Significant differences were found in age groups, marital status, chronic disease groups, BMI, and SUA (*p* < 0.05). However, sex, education level, current residence, smoking status, alcohol consumption, social interaction, and physical activity did not show statistically significant differences between these groups (with and without T2DM) (*p* > 0.05).

Table 4 shows the baseline characterization classified according to quartiles of baseline SUA in 2011 based on the onset of T2DM in 2015. The total number of participants was 5,479, with 1,371, 1,375, 1,365, and 1,368 in quartiles Q1 to Q4, respectively. Significant differences were found in all groups except marital status, physical activity, and chronic disease groups (*p* > 0.05).

Table 5 presents the baseline characteristics classified according to quartiles of relative changes in SUA (changes in SUA from 2011 to 2015). The total number of participants was 5,479, and there were 1,369, 1,371, 1,369, and 1,370 in quartiles Q1 to Q4, respectively. Significant differences were found in sex, marital status, smoking status, alcohol consumption, BMI, and T2DM (*p* < 0.05). By contrast, age, education level, current residence, social interaction, physical activity, and chronic disease groups did not show statistically significant differences between the groups with and without T2DM (*p* > 0.05).

Table 6 displays the baseline characterization classified according to quartiles of absolute changes in SUA (changes from 2011 to 2015). The total number of participants was 5,479, and there were 1,370, 1,370,1,369, and 1,370 in quartiles Q1 to Q4, respectively. Significant differences were observed in age, marital status, and BMI (*p* < 0.05). However, sex, education level, current residence, smoking status, alcohol consumption, social interaction, physical activity, chronic disease groups, and T2DM did not show statistically significant differences between participants with and without T2DM (*p* > 0.05).

The cross-sectional association between SUA at baseline and the prevalence of T2DM in 2011 is presented in Table 7. Without adjusting for covariates, compared with the lowest quartile, the highest quartile of SUA at baseline in 2011 was significantly associated with the prevalence of T2DM in model 1 for females, and all participants, respectively (Q4 vs. Q1) [OR = 1.383 (1.077, 1.775)], [OR = 1.255 (1.047, 1.505)] (*p* < 0.05).

Table 8 shows the prospective association between the SUA baseline in 2011 and the incidence of T2DM in 2015. Compared with the lowest quartile of baseline SUA in 2011, there was no association with the incidence of T2DM from models 1 to 4 in males. Among females, without adjusting for covariates, there was a significant association with the increased incidence of T2DM in model 1 (Q3 vs. Q1), (Q4 vs. Q1) [OR = 1.577 (1.137, 2.189)], [OR = 2.231 (1.631, 3.050)] (*p* < 0.05). After adjusting for age, sex (total subgroup), education level, marital status, and current residence, there was a significant association with incident T2DM in model 2 (Q3 vs. Q1), (Q4 vs. Q1) [OR = 1.523 (1.095, 2.117)], [OR = 2.090 (1.523, 2.867)] (*p* < 0.05) among females. After adjusting for all covariates except BMI, there was a significant association with incident T2DM in model 3 (Q3 vs. Q1), (Q4 vs. Q1) [OR = 1.504 (1.081, 2.092)], [OR = 2.075 (1.511, 2.849)] (*p* < 0.05). This trend was also observed in model 4 (Q4 vs. Q1) [OR = 1.707 (1.234, 2.362)], after adjusting for all covariates (*p* < 0.05). Among all participants, without adjusting for covariates, there was a significant association with the increased incidence of T2DM in model 1 (Q4 vs. Q1) [OR = 1.601 (1.277, 2.008)] (*p* < 0.05). After adjusting for age, sex (total subgroup), education level, marital status, and current residence, there was a significant association with the incidence of T2DM in model 2 (Q4 vs. Q1) [OR = 1.519 (1.204, 1.915)] (*p* < 0.05) among all participants. After adjusting for all covariates except BMI, there was a significant association with incident T2DM in model 3 (Q4 vs. Q1) [OR = 1.597 (1.257, 2.027)] (*p* < 0.05). This trend was also observed in model 4 (Q4 vs. Q1) [OR = 1.380 (1.083, 1.760)] after adjusting for all covariates (*p* < 0.05).

Table 9 presents the association between relative changes in SUA from 2011 to 2015 and the incidence of T2DM in 2015. Compared with the lowest quartile of relative changes in SUA, there were no associations with incident T2DM among males in models 1 to 4. Among females, without adjusting for covariates, there was a significant association with the increased incidence of T2DM in model 1 (Q4 vs. Q1) [OR = 1.409 (1.050, 1.890)] (*p* < 0.05). After adjusting for age, sex (total subgroup), education level, marital status, and current residence, there was a significant association with the incidence of T2DM in model 2 (Q4 vs. Q1) [OR = 1.433 (1.067, 1.926)] (*p* < 0.05). After adjusting for all covariates except BMI, there was a significant association with the incidence of T2DM in model 3 (Q4 vs. Q1) [OR = 1.420 (1.056, 1.910)] (*p* < 0.05). Among all participants, without adjusting for covariates, there were significant associations with the increased incidence of T2DM in model 1 (Q4 vs. Q1) [OR = 1.383 (1.112, 1.720)] (*p* < 0.05). After adjusting for age, sex (total subgroup), education level, marital status, and current residence, there was a significant association with the incidence of T2DM in model 2 (Q4 vs. Q1) [OR = 1.405 (1.129, 1.749)] (*p* < 0.05). After adjusting for all covariates except BMI, there was a significant association with the incidence of T2DM in model 3 (Q4 vs. Q1) [OR = 1.417 (1.138, 1.765)] (*p* < 0.05). This trend was also observed in model 4 (Q4 vs. Q1) [OR = 1.346 (1.079, 1.680)] (*p* < 0.05) after adjusting for all covariates.

Table 10 shows the association between relative and absolute SUA changes and the incidence of T2DM after stratified analysis according to age and BMI. After adjusting for all covariates except age, there were statistically significant differences in incident T2DM for relative changes in the age group of 65–74 years in Q2 [OR = 1.665 (1.014, 2.735)] and Q4 [OR = 1.760 (1.078, 2.873)] (*p* < 0.05). For absolute changes, this trend was also observed in Q4 [OR = 1.949 (1.194, 3.180)] (*p* < 0.05). Furthermore, the relative change in BMI of 18.5–24 kg/m^2^ in Q4 [OR = 1.451 (1.049, 2.003)] (*p* < 0.05) was statistically significant after adjusting for all covariates except BMI.

Table 11 presents the results of interaction analyses between BMI and SUA, age and SUA, and sex and SUA. For the relative changes in SUA, after adjusting for all the covariates, the interaction analysis of SUA and BMI showed a significant difference in the incidence of T2DM for both males and females in model d [OR = 1.054 (1.012, 1.098); OR = 1.081 (1.040, 1.123)] (*p* < 0.05); after adjusting for all covariates, the interaction analysis between age and SUA showed a significant difference in the incidence of T2DM only in females in model g [OR = 1.086 (1.034, 1.140)] (*p* < 0.05). Additionally, after adjusting for all covariates, the interaction analysis between sex and SUA revealed a significant difference in the incidence of T2DM in model h [OR = 1.069 (1.018, 1.122)] (*p* < 0.05). For the absolute changes in SUA levels, the interaction analysis between SUA and BMI showed a significant difference in the incidence of T2DM in females in model d after adjusting for all the covariates [OR = 1.276 (1.107, 1.477)] (*p* < 0.05). Finally, after adjusting for all covariates, the interaction analysis of age and SUA showed a significant difference in the incidence of T2DM only in females in model g [OR = 1.268 (1.063, 1.514)] (*p* < 0.05).

**Table 11:** Odds ratios (ORs) and 95% confidence intervals (CIs) of the interaction analysis with the incidence of T2DM in 2015.

	Subgroups	Relative changes in SUA				Absolute changes in SUA			
BMI*SUA		Model a OR (95% CI)	Model b OR (95% CI)	Model c OR (95% CI)	Model d OR (95% CI)	Model a OR (95% CI)	Model b OR (95% CI)	Model c OR (95% CI)	Model d OR (95% CI)
	Male, *N* = 2,478	1.047 (1.005,1.090)^*^	1.051 (1.009,1.094)^*^	1.055 (1.012, 1.099)^*^	1.054 (1.012, 1.098)^*^	1.048 (0.958,1.148)	1.059 (0.966, 1.160)	1.061 (0.968,1.163)	1.060 (0.967,1.162)
	Female, *N* = 3,001	1.080 (1.040,1.121)^*^	1.085 (1.045,1.127)^*^	1.083 (1.043, 1.125)^*^	1.081 (1.040, 1.123)^*^	1.261 (1.096,1.451)^*^	1.293 (1.123,1.489)^*^	1.287 (1.117,1.483)^*^	1.276 (1.107,1.477) ^*^
Age*SUA		Model a OR (95% CI)	Model e OR (95% CI)	Model f OR (95% CI)	Model g OR (95% CI)	Model a OR (95% CI)	Model e OR (95% CI)	Model f OR (95% CI)	Model g OR (95% CI)
	Male, *N* = 2,478	1.031 (0.985, 1.078)	1,030 (0.985,1.077)	1.032 (0.987,1.079)	1.032 (0.986,1.080)	1.094 (0.945, 1.267)	1.098 (0.948, 1.272)	1.100 (0.950,1.274)	1.109 (0.956,1.285)
	Female, *N* = 3,001	1.107 (1.056, 1.161)^*^	1.098 (1.047, 1.151)^*^	1.090 (1.040, 1.144)^*^	1.086 (1.034,1.140)^*^	1.328 (1.120,1.575)^*^	1.291 (1.089,1.531)^*^	1.266 (1.067,1.503)^*^	1.268 (1.063,1.514)^*^
Sex*SUA		Model a OR (95% CI)	Model b OR (95% CI)	Model d OR (95% CI)	Model h OR (95% CI)	Model a OR (95% CI)	Model b OR (95% CI)	Model d OR (95% CI)	Model h OR (95% CI)
	Total, *N* = 5,479	1.082 (1.032,1.135)^*^	1.088 (1.037,1.141)^*^	1.085 (1.035,1.139)^*^	1.069 (1.018,1.122)^*^	1.123 (0.979, 1.289)	1.153 (1.005,1.323)^*^	1.143 (0.996, 1.312)	1.125 (0.978, 1.294)

^*^p < 0.05.

ORs, odds ratios; CIs, confidence intervals; SUA, serum uric acid; T2DM, type 2 diabetes mellitus; BMI, body mass index.

Model a: unadjusted.

Model b: adjusted for age, education level, marital status, and current residence.

Model c: adjusted for age, education level, marital status, current residence, smoking status, alcohol consumption, social interaction, and physical activity.

Model d: adjusted for age, education level, marital status, current residence, smoking status, alcohol consumption, social interaction, physical activity, and chronic disease.

Model e: adjusted for education level, marital status, and current residence.

Model f: adjusted for education level, marital status, current residence, smoking status, alcohol consumption, social interaction, physical activity, and chronic disease.

Model g: adjusted for education level, marital status, current residence, smoking status, alcohol consumption, social interaction, physical activity, chronic disease, and BMI.

Model h: adjusted for age, education level, marital status, current residence, smoking status, alcohol consumption, social interaction, physical activity, chronic disease, and BMI.

## Discussion

In the cross-sectional study, we found that the baseline SUA was not associated with the prevalence of T2DM for the male, female, and total participants (Table 7), consistent with previous studies (2, 25, 28). In the cohort study, we observed a positive correlation between baseline SUA and a higher incidence of T2DM after 4 years of follow-up (Table 8). Compared with the lowest quartile, participants with the highest SUA quartile level had 1.380-fold increased odds of developing T2DM after adjusting for all covariates. The meta-analyses conducted by Lv et al. (14) provided strong evidence that SUA is considered a potential risk factor for the development of T2DM, with every 1 mg/dL of increase in SUA associated with a 6% increment in the risk of developing T2DM. Another meta-analysis that enrolled 11 cohort studies confirmed the positive association between higher SUA levels and incident T2DM (40). Cheng et al. found that among females the highest quartile had 1.36-fold odds of incident T2DM after 4.5 years of follow-up, which was similar to the incidence observed in our study (20). Additionally, we found that relative changes in SUA levels from 2011 to 2015 were positively correlated with a higher incidence of T2DM after 4 years of follow-up. Compared with the lowest quartile, we found that individuals in the highest quartile of relative changes in SUA levels had 1.346-fold odds of incident T2DM after adjusting for all covariates. Four studies have assessed the association between changes in SUA levels and the incidence of T2DM (2, 25, 28, 29). Tian et al. showed that participants with a higher accumulation of SUA had 1.36-fold increased odds of developing T2DM after 6.99 years of follow-up (28). Moreover, Su et al. demonstrated that the changes in SUA from baseline to 1 year were strongly associated with the incidence of T2DM (OR = 1.30, 95% CI: 1.01, 1.79) (2). Furthermore, Lou et al. obtained the same conclusion after a median follow-up of 3.09 years (OR = 1.93, 95% CI: 1.27, 2.93) (25). These studies suggested that the accumulation and cumulative exposure of SUA are independent predictor factors for T2DM, especially when mediated by the time course. This mechanism may explain the positive association between changes in SUA and incident T2DM after 4 years of follow-up among all participants (2, 25, 28).

The potential mechanism to explain the association between increasing levels of SUA and a higher risk of T2DM has not been fully elucidated. Several hypotheses have been proposed to explain the findings. First, high levels of SUA may cause insulin resistance, a key factor in mediating the progression of the early stage of T2DM (41, 42). The mechanism of interaction between insulin resistance and hyperuricemia may be explained by the phosphorylation of insulin receptor substrate 1 and the increasing oxidase activity of xanthine and nicotinamide adenine dinucleotide phosphate (43, 44). Second, increasing levels of SUA can suppress endothelial nitric oxidative bioavailability and cause endothelial dysfunction (12), which contributes to the progression of oxidative stress, resulting in diabetes being an important part of the pathological mechanism. SUA promotes the production of reactive oxygen species activating pro-inflammatory factors and decreasing nitric oxide bioavailability, thereby inhibiting glucose transporter type 4 translocation and glucose uptake processes (45). Third, higher levels of SUA can impair the cells of the pancreas, damage the function of pancreatic β cells, reduce insulin secretion, and result in metabolic disorders (46, 47). Rocić et al. (48) found that SUA inhibits insulin secretion by binding to an important arginine residue in pancreatic β cells. Additionally, a series of studies have shown that an increase in glucose levels promoted SUA reabsorption through the increased expression of uric acid transporter protein-1 as a compensatory mechanism, and fasting serum insulin compensates for the deficiency of insulin secretion caused by insulin resistance (20, 49). Previous experimental studies have also confirmed that increasing insulin levels promote the involvement of purine nucleoside phosphorylase and xanthine dehydrogenase in uric acid composition (50, 51). In summary, these studies have demonstrated that SUA is implicated in the pathological progression of T2DM, which includes insulin resistance, endothelial dysfunction, and the deterioration of pancreatic β cell function (20, 46, 47). High levels of SUA increase the incidence of T2DM, and high levels of glucose promote the reabsorption of SUA and the progression of T2DM, thus forming a vicious cycle of high levels of SUA and hyperglycemia. Therefore, understanding the interaction between SUA and T2DM may provide another pathway to reduce the potential risk factors for T2DM (52, 53).

Furthermore, most studies have supported a positive association between baseline SUA and the prevalence of T2DM (2, 28, 29, 40, 41). However, some studies reported an inverse correlation between high levels of SUA and T2DM (17, 54, 55). These discrepancies may be attributed to sample size and population selection. Another possible pathological explanation is that hyperglycemia inhibits the proximal tubule reabsorption of SUA; glucose transporter type 9 is a protein that transports uric acid from the lumen to the proximal tubule and is influenced by glucose levels (15, 56–58). Intriguingly, some epidemiological studies from China have reported an L-shape, U-shape, inverted U-shape, and bell-shape relationship between SUA and the pathology of T2DM (15–18), which showed completely different associations with the risk of T2DM.

After conducting a stratified analysis by sex, the national cohort study showed that the association between greater changes in SUA levels and T2DM was even more pronounced in females. For baseline SUA in 2011, we found that females with the highest SUA level had 1.707-fold increased odds of T2DM incidence after adjusting for all covariates. Nevertheless, for relative changes in SUA, although no statistically significant difference was found in model 4, the trend was still evident in models 1, 2, and 3, as shown in Table 9. Previous studies have confirmed this positive correlation, especially among women in China (20, 23, 25, 59, 60). In Korea and Japan, several studies revealed that the increasing level of SUA was strongly related to the risk of T2DM solely among women (61–63). The accumulating evidence clarifies that as a protective hormone, estrogen declines as women go through menopause, leading to a higher level of SUA, impairing endothelial function and causing insulin resistance. The protective function of estrogen gradually disappears, resulting in the eventual onset of diabetes (25, 64). Another study indicated that SUA might induce the β cell dysfunction only in females, suggesting a close association between the higher SUA level and T2DM (58). Additionally, hormone differences between women and men may explain the sex-specific disparity in the correlation between SUA levels and the risk of T2DM. Factors such as the duration of the reproductive period, age at menopause, and use of oral contraceptive pills can affect the level of SUA. Moreover, animal models have shown that sex differences play a regulatory role in controlling glucose homeostasis and the progression of diabetes (65). From a genetic perspective, the gene *SLC2A9* regulates the correlation between SUA and T2DM in women more than in men (24, 66). By contrast, three cohort studies conducted in America, Australia, and the Netherlands, revealed a positive association between higher SUA levels and the incidence of T2DM only among men, not women (57, 67, 68). We hypothesize that sex-specific differences may originate from the differences in sample selection; hence, SUA levels can predict the onset of T2DM in Asians only among females (25).

No correlation was found between high levels of SUA and incident T2DM in men because of differences in fat distribution between women and men (Tables 7–9), which is consistent with a Japanese study (62). In addition, some studies have demonstrated that there was no relationship between SUA and T2DM among the public and male workers, which is consistent with our study (69, 70). By contrast, other studies have shown that in men, high SUA levels are related to a decreased risk of T2DM (27, 41). The authors explained that the possible reasons for the protective effect may be that higher SUA levels can reduce oxidative stress levels and delay impaired glucose tolerance (71). Furthermore, the difference between the sexes may be a result of the role of estrogen (26). Another possible mechanism explaining the inverse association is that hyperglycemia eventually exceeds the glucose threshold of the kidney, leading to glucose excretion in the urine. As a high-volume urate transporter protein, glucose transporter protein 9 mediates urate efflux through the proximal tubular apical membrane, thereby inhibiting uric acid reabsorption, enhancing uric acid excretion, and reducing serum uric acid levels (22, 56, 57). Additionally, owing to the limited sample size, some previous studies regarding the association between high-level SUA and the risk of T2DM lacked stratification by sex, leading to varying conclusions. Furthermore, the participants were often restricted to certain groups of people, including workers or patients, which may be another reason for the discrepancy (72, 73).

The association between relative changes in SUA with the incidence of T2DM in 2015 was greatly attenuated after adjusting for BMI (Table 9), suggesting that the association was partially mediated by BMI. Most studies supported the positive association between BMI and T2DM; however, the cutoff points differ among these studies. One study suggested that individuals with a BMI of ≥24 kg/m^2^ had a higher risk of developing T2DM (21), while another study indicated that individuals with a BMI of ≥28 kg/m^2^ had a higher risk of developing T2DM (74). This positive association between SUA and BMI may be explained by the fact that a higher BMI is strongly associated with obesity, and BMI can act as a mediator between SUA and diabetes mellitus, potentially affecting the incidence of T2DM (52, 53). Conversely, a study by Qiu et al. indicated that individuals with a BMI of <28 kg/m^2^ were prone to T2DM (75). In our study, we found that individuals with a BMI of 18.5–24 kg/m^2^ were more prone to developing T2DM (Table 10). Additionally, the participants aged 65–74 years had the highest risk of developing T2DM after adjusting for all covariates. The mechanisms underlying these findings are unclear, and additional cohort studies are needed to explore the relationship between SUA and T2DM risk. Some samples were excluded due to a lack of complete data records; hence, complete data should be collected for further studies.

In our study, we explored the interactions between SUA and BMI, age and SUA, and sex and SUA. We found that BMI and the relative changes in SUA levels had a combined effect on the incidence of T2DM among females and males, suggesting that BMI was an important mediator of the progression of SUA. Evidence has shown that an increased BMI is often accompanied by obesity, and the resulting accumulation of visceral fat produces large amounts of free fatty acids, leading to an increased production of uric acid (76). Additionally, we found that age and the changes in SUA had a combined effect on the incidence of T2DM only among females, which supported the notion that females were more prone to develop T2DM after menopause, consistent with a previous study (77). Furthermore, the combined effect of sex and SUA was strongly associated with the incidence of T2DM, suggesting that females were more prone to develop T2DM than males, which was in line with the results presented in Table 8.

### Strengths and limitations of the study

This study had several strengths. First, we conducted a cross-sectional and prospective study simultaneously to reveal the association of SUA at baseline and changes in SUA levels with the incidence of T2DM. Second, the sex difference was assessed systematically based on different parameters with the incidence of T2DM. Additionally, stratified analysis by age and BMI was conducted using the quartile method, which revealed the relationship between the concentration of SUA and the incidence of T2DM. Moreover, we performed an interaction analysis of BMI, age, sex, and the changes in SUA.

Nevertheless, this study had certain limitations. First, we did not collect relevant information regarding dietary habits and medication use, including the frequency and type of high-purine food intake and medications that affect uric acid metabolism. Thus, the study did not exclude these important confounders. Second, the effect of SUA on the incidence of T2DM was only collected at one time point and value of change in this study. A more comprehensive evaluation of the effect of SUA on the incidence of T2DM could be obtained if SUA values from multiple years were included. Third, some samples were excluded due to a lack of complete data records; hence, complete data should be collected for further studies.

## Conclusion

Our cross-sectional study revealed no association between SUA baseline and the prevalence of T2DM in 2011. However, after 4 years of follow-up, we found a positive correlation between the SUA baseline and the incidence of T2DM in 2015, as well as the relative change in SUA from 2011 to 2015, indicating the cumulative contribution effect of SUA. Additionally, the stratified analysis by sex, age, and BMI revealed a sex-specific association between SUA and the incidence of T2DM. In summary, hyperuricemia is a progressive process, and our findings suggest that hyperuricemia exposure duration is positively associated with the risk of T2DM. Therefore, early intervention and treatment of high levels of SUA may play a vital role in reducing the incidence of T2DM.

## Data availability statement

The original contributions presented in the study are included in the article/supplementary material, further inquiries can be directed to the corresponding author. The data can be obtained at http://opendata.pku.edu.cn/dataverse/CHARLS.

## Ethics statement

The studies involving human participants were reviewed and approved by the Medical Ethical Board of Wannan Medical College (approval number 2021-3). The patients/participants provided their written informed consent to participate in this study.

## Author contributions

LZ: conception and design. CW: wrote the manuscript. LZ and CW: data analysis. CW, LZ, RW, JW, TY, DZ, LY, MW, HL, HW, JL, YL, LS, YH, ML, and XL: revised and reviewed the manuscript. All authors contributed to the article and approved the submitted version.

## Funding

This study was supported by the NSFC (70910107022 and 71130002), National Institute on Aging (R03-TW008358-01; R01-AG037031-03S1), World Bank (7159234), the Program for Outstanding Young Talents from the Universities and College of Anhui Province for LZ (gxyqZD2021118), Anhui Education Department Foundation (SK2019A0223), and Wannan Medical College Foundation for Teaching Research Project (2020jyxm45).

## Conflict of interest

The authors declare that the research was conducted in the absence of any commercial or financial relationships that could be construed as a potential conflict of interest.

## Publisher’s note

All claims expressed in this article are solely those of the authors and do not necessarily represent those of their affiliated organizations, or those of the publisher, the editors and the reviewers. Any product that may be evaluated in this article, or claim that may be made by its manufacturer, is not guaranteed or endorsed by the publisher.

## Abbreviations

SUA: serum uric acid
T2DM: type 2 diabetes mellitus
CHARLS: China Health and Retirement Longitudinal Study
CAPI: Computer Assisted Personal Interview
BMI: body mass index
ANOVA: analysis of variance
OR: odds ratio
CIs: confidence intervals
SD: standard deviation
FPG: fasting plasma glucose
HbA1c: glycated hemoglobin NADPH nicotinamide adenine dinucleotide phosphate
NO: nitric oxide
ROS: reactive oxygen species
GLUT4: Glucose transporter type 4
